# Associations Between Structural and Social Determinants of Health With COVID Infection Rates at a Safety Net Hospital

**DOI:** 10.7759/cureus.17397

**Published:** 2021-08-23

**Authors:** Dotun Ogunyemi, Rolando Mantilla, Abhinav Markus, Aubrey Reeves, Suyee Win, Devin R Barrientos, Dandrich Lim, David Lanum, Niren Raval

**Affiliations:** 1 Obstetrics and Gynecology, Arrowhead Regional Medical Center, Colton, USA; 2 Healthcare Program/Population Health, Arrowhead Regional Medical Center, Colton, USA; 3 Family Medicine, Arrowhead Regional Medical Center, Colton , USA; 4 Family Medicine, California University of Science and Medicine, Colton, USA; 5 Family Medicine, Arrowhead Regional Medical Center, Colton, USA

**Keywords:** covid-19, healthcare disparities, infection rates, southern california, minorities, community medicine, infection rate

## Abstract

Background: The reported disproportionate impact of COVID-19 infections on minority populations may be due to living in disinvested communities with a high level of poverty, pollution, inadequate unsafe employment, and overcrowded housing.

Objective: To determine the association of county, city, and individual risk factors with COVID-19 infection rates.

Methods: Retrospective chart review on COVID-19 tests performed from March through July 2020 at Arrowhead Regional Medical Center (ARMC), Colton, California.

Results: A total of 7104 tests were performed with 69% in the drive-through testing center. The mean duration of test-to-results time was 2.36 (+0.02) days. COVID-19 positive tests occurred in 1095 (15.4%). At least one symptom occurred in 414 (33%) with a sensitivity of 37.8, specificity of 86.02, a positive predictive value of 33.01, and a negative predictive value of 72.76. Individual factors significantly associated with testing positive for COVID-19 were diabetes, Hispanic ethnicity, and male gender. Younger age was significantly associated with testing COVID positive with the highest risk in children <10 years.

COVID-19 positive persons significantly resided in cities with higher population density, household members, poverty, non-English speaking homes, disability, lower median household income, lack of health insurance and decreased access to a computer and Wi-Fi services. County health rankings showed a significant positive association between testing positive for COVID-19 with increased smoking, air pollution, violent crimes, physical inactivity, decreased education, and access to exercise.

Conclusion: Adverse county health rankings, socially and economically disadvantaged cities are associated with an increased risk of testing positive for COVD-19. This information can be used in strategic planning and invention mitigation.

## Introduction

Severe acute respiratory syndrome coronavirus 2 (SARS-CoV-2) was first reported in the United States in January 2020. Since then, nearly 18 million cases of SARS-CoV-2 infection with over 300,000 deaths have been reported in the US and is still rising [1]. Currently, high vaccination coverage among the general population in addition to individual prevention measures and social distancing are critical to preventing COVID-19 infections in the United States [2,3]. Reports have suggested that African American and Hispanic populations are disproportionately impacted by COVID-19 disease [4,5]. Social and structural determinants of health could potentially explain some of the racial disparities observed with COVID-19 infection. Understanding the reason for disparities among different populations is crucial for developing interventions to prevent transmission of COVID-19 infections. Previous studies investigating health care disparities in COVID-19 infections have focused less on infection transmission but more on morbidity and mortality with studies mainly from the Northeast United States and other countries [6-9]. Thus, there is a need for investigations from western United State populations on correlations between COVID-19 infection rates with health care disparities and socio-structural health determinants. Consequently, the objective of this study was to evaluate the associations between COVID-19 test results with individual-level comorbidity and demographic factors, city-level health determinant factors and county-level health care rankings from a single institution in southern California.

The article was posted as a preprint in Research Square on March 10, 2021 (https://www.researchsquare.com/article/rs-296897/v1).

## Materials and methods

This is an IRB-approved retrospective study of persons who had a COVID-19 test performed from March 1, 2020, to July 31, 2020, at Arrowhead Regional Medical Center (ARMC) in Colton, California. ARMC is a 456-bed county hospital which is the tertiary safety net hospital for San Bernardino County. San Bernardino County is the largest county in the contiguous United States by area and is slightly larger than the states of New Jersey, Connecticut, Delaware, and Rhode Island combined. The population estimate from July 1, 2019, was 328,239,523, which comprised of Hispanics 60.8%, non-Hispanic Whites 32.8%, Asian 3.9%, and African American 1.6% [10]. ARMC provides emergency, primary, and specialty care to the community and has more than 275,000 visits annually in both inpatient and outpatient settings. ARMC is designated as a level II trauma hospital and is the regional burn center covering the largest landmass in the United States.

The electronic records of 7104 people who had either outpatient or inpatient COVID-19 testing using the polymerase chain reaction (PCR) laboratory technique were extracted for analysis. Individual data obtained for analysis included gender, race, age, primary spoken language, sexual orientation, incarceration, homelessness, and current address. Medical comorbidities included body mass index, substance abuse, mental disease, asthma, chronic obstructive airway disease (COPD), hypertension, and diabetes. Presumed COVID-19 symptoms collected were new-onset fever, cough, shortness of breath, fatigue/weakness, anosmia, sore throat, body aches, nausea, and vomiting.

We used each person’s reported address to assign them into cities and counties. We obtained city data from United States Census Bureau: Quick Facts United States database [10]. The data on cities used for analysis included city population, number of household members, percentages of non-English speaking people, high school graduates, bachelor’s degree or higher, people with a disability, access to health insurance, a household with a computer, with broadband internet and people living in poverty. We also obtained for each city the median household income in 2019 dollars and population density per square miles.

All addresses obtained were assigned to the appropriate county. We obtained county-level health rankings from the 2020 County Health Rankings program collaboration between the Robert Wood Johnson Foundation and the University of Wisconsin Population Health Institute [11]. Health county rankings analyzed adults smoking, air pollution, high school graduation, college graduate, violent crimes, access to exercise, physical inactivity, and water violations.

Models were generated using Statistical Package for Social Sciences (SPSS) version 26 software (IBM Corp., Armonk, New York) to determine the relationship between individual, city and county-level risk factors and COVID-19 test results. For univariate analysis, ANOVA, student T-test, and Chi-square tests were performed to see associations between COVID-19 test results and risk factors. Variables were entered into a logistic regression model to determine, independently, significant associations.

Because of the many statistical tests conducted, a more conservative P-value of 0.01 or less was taken as significant to reduce and adjust for type 1 errors. All data analysis was conducted using IBM SPSS Statistics 26.0. The study was approved by the institutional IRB.

## Results

A total of 7104 persons had the COVID-19 PCR test performed at ARMC from March and July 2020. Of these, 4926 (69%) were performed an outpatient testing category done on the hospital campus as an extension of public health effort by the county of San Bernardino, 1579 (22.2%) in the emergency department, 301 (4.2%) in ancillary sites such as Arrowhead Family Centers, 130 (1.8%) on inpatient services, and 167 (2.4%) at unknown sites. In March 2020, 167 (2.3%) tests were performed, 848 (11.9%) in April 2020, 2177 (37.8%) in May 2020, 2683 (37.8%) in June 2020, and 1235 (17.4%) in July 2020. The mean time interval for testing-to-results was 2.36 days (standard error of the mean [SEM] +0.002), median time was two days with a range of 0-14 days.

**Figure 1:**
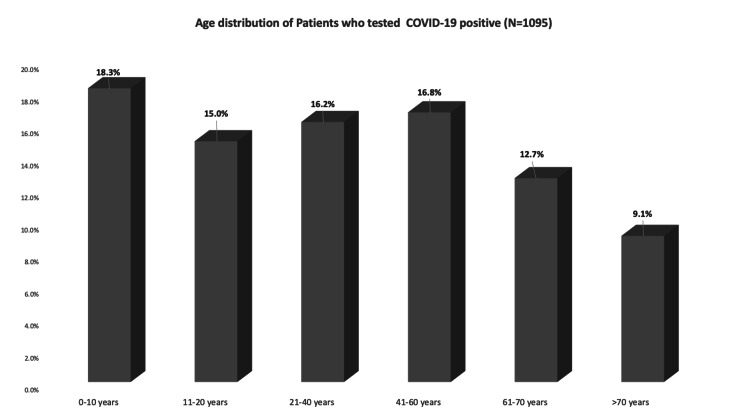
Age distribution associations with COVID-19 test results Chi-square analysis performed: P<0.001. The population was divided into six quartiles (ages in years: 0-10, 11-20, 21-40, 41-60, 61-70, and >70), and testing COVID negative (N=6009) and COVID positive (N=1095) were analyzed for each quartile. In ages 0-10 years, 210 (rate of 81.7%) tested COVID negative and 47 (rate of 18.3%) tested COVID positive. In ages 11-20 years, 438 (rate of 85%) tested COVID negative and 77 (rate of 15%) tested COVID positive. In ages 21-40 years, 2250 (rate of 83.8%) tested COVID negative and 435 (rate of 16.2%) tested COVID positive. In ages 41-60 years, 1841 (rate of 83.2%) tested COVID negative and 372 (rate of 16.8%) tested COVID positive. In ages 61-70 years, 820 (rate of 87.3%) tested COVID negative and 119 (rate of 12.7%) tested COVID positive. In ages >70 years, 450 (rate of 90.9%) tested COVID negative and 45 (rate of 9.1%) tested COVID positive. Mean was found to be 43.12 for COVID negative and 40.76 for COVID positive.

Analysis of individual risk factors showed that diabetes comorbidity had the highest odds ratio (OR) 3.6, for testing positive for COVID-19. This was followed by hypertension (OR= 2.7), Hispanic ancestry or Spanish as a primary language (OR=2.6), and male gender (OR of 1.2). African American, Asian, or non-Hispanic White ancestry were all associated with a decreased risk of testing positive for COVID-19 as seen in Table 1. The mean age of persons who tested positive for COVID-19 was younger than those who tested negative (40.76 versus 43.12 years, P<0.001). Figure 1 shows the distribution of the age of COVID-19 positive persons with children <10 years having the highest rate (18.3%), with a lower rate in the age group of 61-70 years (12.7%) and the lowest rate in those >70 years of age (9.1%).

**Table 1:** Significant negative and positive demographic/comorbid associations with COVID-19 test results in 7014 persons tested at Arrowhead Regional Medical Center, Colton, California between March and July 2020 Chi-square tests were performed to determine significant associations of COVID-19 positive testing. Positive associations are identified with an odds ratio >1 and those with a negative association have an odds ratio <1. The data are arranged from largest to lowest odd ratio.

	Demographic/comorbid factors present	Demographic/comorbid factors absent	P-value	Odd ratio
Positive associations				
Risk factor	Diabetes	No diabetes		
COVID positive	70 (30.4%)	64 (10.9%)	<0.001	3.6
Risk factor	Hypertensive	Not Hypertensive		
COVID positive	47 (29.4%)	87 (13.2%)	<0.001	2.7
Risk factor	Hispanic	Non-Hispanic		
COVID positive	862 (19.9%)	208 (8.6%)	<0.001	2.6
Risk factor	Spanish speaking	English speaking		
COVID positive	232 (30%)	698 (14.2%)	<0.001	2.6
Risk factor	Male	Female		
COVID positive	451 (17.6%)	512 (15.2%)	<0.001	1.2
Negative associations				
Risk factor	Asian	Non-Asian		
COVID positive	28 (9.7%)	1067 (15.7%)	<0.006	0.6
Risk factor	African American	Non-African American		
COVID positive	59 (9.7%)	1011 (16.5%)	<0.001	0.5
Risk factor	White	Non-Hispanic White		
COVID positive	97 (7.9%)	973 (17.6%)	<0.001	0.4

Overall, 414 (33%) persons of those who tested positive for COVID-19 had any symptoms with sensitivity, specificity, positive predictive value, and negative predictive value, respectively, of 37.8, 86.02, 33.01, and 72.76. Of the symptoms of cough, fever, and dyspnea; cough had the highest while fever had the lowest sensitivity and specificity as seen in Table 2.

**Table 2:** Correlations between COVID-19 symptoms and COVID-19 test results in 1095 persons who tested positive and 6009 persons who tested negative OR: odds ratio.

Symptoms	COVID+	COVID−	P-value (OR)	Sensitivity	Specificity	Negative predictive value	Positive predictive value
Any symptom	414 (33%)	840 (67%)	<0.001 (3.74)	37.8	86.02	33.01	72.76
Cough	313 (38.8%)	493 (61.2%)	<0.001 (4.48)	28.58	91.7	38.83	87.58
Fever	279 (41.7%)	390 (58.3%)	<0.001 (4.9)	25.48	87.32	41.7	87.31
SOB	186 (29.2%)	452 (70.8%)	<0.001 (2.52)	17	92.48	29.15	85.94

**Figure 2:**
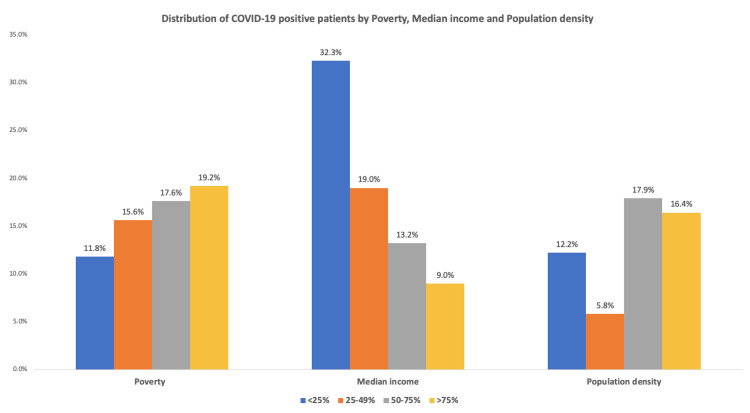
Distribution of COVID positive patients by quartiles for poverty, median income, and population density* *P<0.001 for all analyses. Patients were divided into four quartiles (<25%, 25–49%, 50–75%, and >75%) for poverty, median income, and population density to analyze testing COVID negative (N=5966) and COVID positive (N=1092). Within the poverty category, in <25% quartile 2337 (rate of 88.2%) tested COVID negative and 313 (rate 11.8%) tested COVID positive. In 25-49% quartile, 949 (rate of 84.4%) tested COVID negative and 176 (rate 15.6%) tested COVID positive. In 50-75% quartile, 1402 (rate of 82.4%) tested COVID negative and 300 (rate 17.6%) tested COVID positive. In >75% quartile, 1278 (rate of 80.8%) tested COVID negative and 303 (rate 19.2%) tested COVID positive. Within the median household income in 2019 in dollars category, in <25% quartile 1519 (rate of 67.7%) tested COVID negative and 353 (rate 32.3%) tested COVID positive. In 25-49% quartile, 1440 (rate of 81%) tested COVID negative and 337 (rate 19%) tested COVID positive. In 50-75% quartile, 2009 (rate of 86.8%) tested COVID negative and 305 (rate 13.2%) tested COVID positive. In >75% quartile, 994 (rate of 91%) tested COVID negative and 98 (rate 9%) tested COVID positive. Within the population density per square mile category, in <25% quartile 1988 (rate of 87.8%) tested COVID negative and 276 (rate 12.2%) tested COVID positive. In 25-49% quartile, 65 (rate of 94.2%) tested COVID negative and 4 (rate 5.8%) tested COVID positive. In 50-75% quartile, 2712 (rate of 82.1%) tested COVID negative and 592 (rate 17.9%) tested COVID positive. In >75% quartile, 995 (83.6%) tested COVID negative and 195 (16.4%) tested COVID positive.

Based on their home addresses, tested individuals were assigned to approximately 40 cities. COVID-19 positive rates ranged from 20% to 5% in 18 cities that had at least 50 citizens tested. San Bernardino and Rialto cities both had the highest rate of 20%, while Redlands and Yucaipa had the lowest rate of 5%. Table 3 reports COVID-19 positive rates and the social and structural determinants of health factors distributions in the cities where the tested persons lived. Table 4 addresses the associations between the social and structural determinants of the cities and testing COVID positive rates. This showed that cities with larger populations, higher household numbers, more non-English speakers, fewer persons with a high school or graduate degree, more persons with disabilities or no health insurance, and households with fewer computers or broadband internet were more likely to test positive for COVID-19. Furthermore, cities with an increased number of persons living in poverty, lower median household income, and more densely populated had higher COVID positive rates as seen in Table 4. For a more detailed analysis, we explored the association with quartile distributions. Figure 2 showed that a higher rate of testing positive for COVID-19 was associated with an increased number of persons living in the poverty quartile and decreased with increasing median income quartile. For population density, the risk of COVID-19 positivity was lowest in the second quartile and highest in the third quartile.

**Table 3:** Social and structural determinants of health factors distributions in cities and COVID-19 positive rates with at least 50 residents tested for COVID-19 at ARMC

City	COVID positive rate	Population	Population density	No of household members	Non-English speaker	High school graduate	College graduate	Persons with disability	Persons with no health insurance	Household computer	Household broadband internet	Median household income in 2019 dollars	Population density per square miles
San Bernardino	19.70%	3546.00	215784.00	3.46	51.20	69.40	11.90	8.90	12.80	90.10	73.40	45834.00	0.26
Rialto	19.60%	4437.00	103526.00	3.94	59.10	70.30	11.10	6.70	13.40	94.60	77.90	72918.00	0.13
Colton	19.30%	3403.40	54824.00	3.25	50.80	76.40	17.30	7.90	8.80	90.60	82.40	61518.00	0.16
Hesperia	19.20%	1233.60	95750.00	3.52	33.60	78.20	11.30	7.10	8.40	93.90	89.40	53838.00	0.15
Bloomington	19.00%	3983.80	23851.00	3.76	66.50	57.70	9.70	8.20	11.80	87.50	73.60	90953.00	0.08
Fontana	17.40%	4620.80	214547.00	3.85	58.10	75.50	18.10	5.40	10.20	95.40	89.50	61602.00	0.15
highland	14.40%	2831.50	55417.00	3.40	41.50	77.30	21.90	6.50	10.30	90.90	79.80	52085.00	0.20
Rancho Cucamonga	12.50%	4147.20	177603.00	3.06	32.80	91.50	35.30	5.40	5.20	96.50	92.30	74839.00	0.11
Loma Linda	12.50%	3094.90	24473.24	2.60	42.24	88.76	44.71	7.02	6.87	89.47	82.57	64868.00	0.18
Ontario	12.20%	3282.40	185010.00	3.48	68.10	73.90	17.10	5.70	11.10	92.50	83.10	104590.00	0.07
Victorville	10.60%	1583.90	122385.00	3.60	38.40	77.70	12.90	9.00	8.10	94.00	87.90	69104.00	0.10
chino	6.50%	2371.05	83853.00	3.19	42.30	93.70	46.70	4.00	4.70	98.30	94.80	72782.00	0.13
Moreno valley	6.50%	3771.10	213055.00	4.06	50.40	76.40	16.30	7.10	11.80	95.20	90.40	72782.00	0.13
Upland	6.50%	4721.30	77140.00	2.82	32.70	89.40	32.40	7.20	6.70	92.40	86.40	53561.00	0.20
grand terrace	6.10%	3438.00	12584.00	2.84	28.90	90.90	26.80	8.50	5.30	92.50	90.10	55607.00	0.17
Redlands	5.80%	1903.00	71513.00	2.79	25.90	88.80	41.80	7.80	5.70	90.60	85.40	71788.00	0.09
Yucaipa	5.40%	1841.90	53921.00	2.92	24.40	89.30	24.40	7.70	5.40	92.90	84.40	66134.00	0.14

**Table 4:** The associations of city-level social and structural determinants of health factors of persons tested with COVID-19 test results () = standard error of the mean; ^!^ = means; 95%CI = 95% confidence interval of the mean difference; social determinant = city of a patient tested obtained from United States Census Bureau: Quick Facts United States [10]. Student T-test and ANOVA were used as indicated for statistical analysis.

Social determinant	COVID negative (5750)	COVID positive (1067)	p-Value	95%CI
City population^!^	140138.04 (1011.5)	147763.65 (2257.3)	0.003	12607.50–2643.71
No of household members^!^	3.46 (0.005)	3.54 (0.01)	<0.001	0.11–0.06
Non-English speaking	48.99% (0.16)	52.11% (0.3)	<0.001	3.90–2.33
High school graduate	76.64% (0.11)	74.08% (0.22)	<0.001	1.99–3.12
Bachelor’s degree or higher	19.63% (0.13)	16.79% (0.24)	<0.001	2.19–3.48
Persons with disability	7.15% (0.02)	7.25% (0.04)	<0.001	0.21–0.00
Persons with no health insurance	10.11% (0.04)	10.85% (0.08)	<0.001	0.93–0.56
Household computer	92.74% (0.04)	92.44% (0.08)	0.001	0.11–0.46
Household broadband internet	82.55% (0.09)	81.01% (0.2)	<0.001	1.09–1.98
Persons in poverty	16.24% (0.02)	17.63% (0.01)	<0.001	0.02–0.01
Median household income in 2019 dollars!	64108.74 (414.1)	60531.82 (182.2)	<0.001	2593.24–4560.61
Population density per square miles	3572.21 (13.45)	3787.90 (25.29)	<0.001	215.69–33.09

The county of residence of the individuals who tested for COVID were 6544 (92.8%) from San Bernardino County, 364 (5.2%) from Riverside County, 99 (1.4%) from Los Angeles County, 24 (0.3%) from Orange County, and 24 from other Californian counties and other states in the Union. The COVID positive rates were as follows: San Bernardino County 15.9%, Riverside County 9.6%, Los Angeles County 9.1%, and Orange County 4.2 %. Table 5 shows the associations between county health outcomes and testing COVID positive. Persons who tested positive for COVID were significantly more likely to come from a county with more adults who smoke, more air pollution, fewer citizens graduating from high school or college, more violent crimes occurrence, less access to exercise opportunities, and increased leisure-time physical inactivity.

**Table 5:** Associations of county health ranking with COVID-19 tests results () = standard error of the mean; 95%CI = 95% confidence interval of the mean difference; County data: data obtained from the website [11]. Violent crime = violent rate crime rate/100,000. Air pollution = air pollution − particulate matter = average daily density of fine particulate matter in micrograms per cubic meter (PM2.5). Adult smoking = percentage of adults who are current smokers. Physical inactivity = percentage of adults aged 20 years and over reporting no leisure-time physical activity. Access to exercise: access to exercise opportunities: percentage of the population with adequate access to locations for physical activity. Student T-test and ANOVA were used as indicated for statistical analysis.

County data	COVID negative (N=5943)	COVID positive (N=1088)	P-value	95%CI
Adult smoking	12.90 (0.005)	12.95 (0.008)	<0.001	0.074–0.023
Air pollution	14.87 (0.003)	14.89 (0.004)	<0.001	0.037–0.003
High school graduation	83.34 (0.018)	83.19 (0.03)	<0.001	0.061–0.024
College graduate	55.18 (0.017)	55.08 (0.026)	0.019	0.016–0.182
Violent crime	433.46 (0.49)	437.31 (0.85)	0.001	6.210–1.507
Access to exercise	84.55 (0.03)	84.29 (0.05)	<0.001	0.120–0.399
Physical inactivity	22.75 (0.001)	22.87 (0.001)	<0.001	0.185–0.052

Table 6 shows the results of significant independent associations from logistic regression analysis. For individual-level factors, male gender, age, Hispanic ethnicity, hypertension, and diabetes were all independently significantly associated with testing positive for COVID-19. For city-level risk factors, non-English speaking, population density, and number of household members were significantly associated with a positive COVID test result while being a college graduate and median household income were independently associated with testing COVID negative. For county risk factors, adult smoking was independently associated with testing COVD positive.

**Table 6:** Significant independent associations of COVID-19 test results by logistic regression analysis For individual risk factors, BMI, African Americans, Asians, and Non-Hispanic Whites were also included in the models but did not reach statistical significance; for city risk factors, variables entered into models that did not reach significance were households with broadband internet, persons with no health insurance, and persons in poverty; for county risk factors, variables entered into the model that did not reach significance were air pollution, county regions. 95% CI=95% confidence interval of the p-value.

Risk factor	Coefficient	P-value	Odds ratio	95% CI
Individual risk factors				
Male gender	0.65	0.003	1.91	1.2–2.9
Age	0.31	0.003	1.36	1.12–1.67
Hispanic	0.73	0.002	2.08	1.32–3.28
Diabetes	0.72	0.002	2.05	1.3–3.25
Hypertension	0.53	0.024	1.70	1.07–2.70
City risk factors				
Non-English speaking	0.01	0.005	1.01	1.004–1.02
College graduate	−0.02	0.004	0.98	0.96–0.99
Population density	0.13	<0.001	1.14	1.07–1.21
Median home income	−0.29	<0.001	0.75	0.64–0.88
No of household members	0.42	<0.001	1.52	1.22–1.90
County risk factors				
Adult smoking	0.60	0.003	1.82	1.22–2.7

## Discussion

Our study focused on the associations of both individual risk factors and socio-structural determinants of health at city and county levels with testing positive for COVID-19 in a tertiary hospital setting in southern California. Most published studies have focused on risk factors for hospitalization and mortality [12]. Nazroo and Becares from the United Kingdom reported that rates of COVID-19-related mortality within a local authority increased as the proportion of the population who were of ethnic minority increased [13]. Goodman et al. in a cross-sectional United States population demonstrated that men, hypertension, obesity, and age 20-39 years were associated with the highest mortality in hospitalized patients [14]. Zhang et al. from the Wuhan epicenter demonstrated that male sex, a severe COVID-19 condition, expectoration, muscle ache, and decreased albumin were independent risk factors for mortality [8]. Currently, COVID-19 vaccination remains the most effective means to achieve control of the pandemic. The vaccination roll-out with the three approved vaccines [Pfizer-BioNTech COVID-19 vaccine (mRNA); Pfizer-BioNTech COVID-19 vaccine (mRNA); Janssen COVID-19 Vaccine (Johnson & Johnson) (viral vector)] have been partly successful but is now plateauing. As of July 2021, about 50% of the American population are fully vaccinated, while the goal of herd immunity is stated to be about 70-80%. However, vaccine hesitancy is now a problem with the new more transmissible and more deadly Delta variant causing an upsurge and a new wave of infections resulting in increased hospitalization and mortality in mainly unvaccinated people. Hence, there is an urgency to get more people vaccinated before a more deadly variant that is resistant to the current vaccine emerges [15]. Thus, our study and similar studies in various local regions identifying regional population-based risk factors that can be targeted by public health and other stakeholders to counteract vaccine hesitancy and facilitate the development of mitigating programs and strategies may be helpful in turning the tide of the pandemic in specific communities [6,9].

Symptoms

Our study showed that of all persons who presented with symptoms, 33% (true positive) tested COVID positive and 67% (false positive) tested COVID negative. Thus, COVID symptoms have very low sensitivity (37.8%), specificity (86%), and positive predictive value (33%). Therefore, in San Bernardino County, symptoms do not seem to be discriminant in identifying those who are likely to test COVID positive. These data are in support of the current strategy of widespread community testing. However, others have noted higher symptomatology, but those studies were mainly from a hospital population. Yang et al. in a meta-analysis of 576 hospitalized patients in China reported clinical symptoms of fever 91.3%, cough 67.7%, fatigue 51.0%, and dyspnea 30.4% [9]. However, in contrast, our study was more community-based and with over 70% of testing occurring in the drive-thru or ancillary sites which would account for our lower rates by the inclusion of asymptomatic no-hospitalized persons. In our study cough was the widespread symptom followed by fever and dyspnea. Very few cases of anosmia were reported. This is in contrast to a report by Speth et al. who reported on 103 patients in Switzerland who reported in COVID-19 positive patients fever 74%, cough 68%, dyspnea 46.6%, and hyposmia/anosmia 61%. However, in this study, patients were contacted by phone and asked specifically about olfactory symptoms [16].

Age

Our data showed that the COVID-19 positive rate of 18% for children <10 years was the highest for all age groups. The susceptibility of children to COVID-19 is controversial. A report from the Chinese CDC revealed that only 1% of COVID-19 infections occurred in children [17]. However, Liu et al. from Guangdong Province China reported that children <10 years had a higher COVID-19 infection rate (5.7%) than contacts aged 10-59 years [18]. Furthermore, our study also revealed that the lowest COVID positive rate was in persons older than 60 years old (12.7% for 61-70 years, 9.1% for >70 years). The United States data reported by CDC at the time of hotspot detection showed that the highest positivity was among persons aged 18-24 years (14%), followed by those aged 0-17 years (11%), 25-44 years (10%), 45-64 years (8%), and ≥65 years (6%) [19]. From China, Lui et al. demonstrated that the highest infection rate was in the group aged 19-60 years, while another recent article in Taiwan did not observe a significantly higher infected risk of elderly contacts [20]. These different findings support the need for local identification of age groups at greatest risk of being infected with COVID in developing locally effective prevention strategies. The finding of a high rate of infections in children in this study has important implications in the ongoing debate on virtual versus in-person schooling from K1-12.

Race

Our study confirmed that Hispanic or Spanish-speaking people were significantly and independently at higher risk for testing positive for COVID-19 compared to African Americans, Asians, or non-Hispanic Whites. Current reports suggest that Black and Hispanic populations are disproportionately impacted by COVID-19 disease with increased rates of infection compared to white populations. It has been postulated that these racial disparities may not reflect an independent predisposition to the disease. In a study of COVID-19 patients in an emergency department, while Black patients were disproportionately represented in the cohort of patients requiring hospital admission or ICU care, the association was no longer evident after adjusting for insurance status and comorbidities [5]. Similarly, in our study, even though on univariate analysis, African Americans appeared to be at lower risk of testing positive for COVID-19, this was no longer evident on regression analysis. The increased risk for the Latino population is supported by a modeling study using U.S. County-level characteristics which revealed that structural factors placed Latino populations and particularly monolingual Spanish speakers at elevated risk for COVID-19 acquisition [21]. Similar to other studies we found that comorbidities of diabetes, hypertension, and adult smoking were associated with testing COVID positive. A possible factor that may play a role in increasing the risk of diabetes and/or obesity is the impaired innate and adaptive immune response, characterized by a state of chronic low-grade inflammation that can lead to increased susceptibility to infection while hypertension may also predispose vascular changes favoring COVID-19 infection [22].

Health inequities

COVID positive rates varied from 5% to 20% between cities and from 4% to 16% between counties. Analysis of social and structural health determinants accounted for all the geographical variations of COVID testing. Geographical areas with lower COVID-19 positivity compared to those with higher COVID-19 positive rates had the tendency to be less overcrowded, more educated, wealthier with more resources, and improved health rankings. Rodriguez-Diaz et al. noted that Latino COVID-19 diagnosis and deaths were associated with crowded living conditions and increased household occupancy density, air pollution, and employment [21]. They discussed that monolingual Spanish speakers are more likely to be occupationally exposed through involvement in factory or service industry jobs such as meatpacking plants and deemed as “essential” or “frontline” workers [19]. Wadhera et al. found out that Bronx New York had the highest proportion of racial/ethnic minorities, the most persons living in poverty, and the lowest levels of educational attainment had higher rates of hospitalization and death related to COVID-19 than the other four boroughs [23]. Analysis of the socioeconomic, demographic, epidemiological factors, and the health system structure of Brazil concluded that 59.8% of the variation in the incidence of COVID-19 in Brazil was as a result of income inequality and high home density. The authors suggested that authorities should implement comprehensive actions to ensure good economic conditions and strengthening health networks for populations with socioeconomic vulnerability.

Limitations

This study has many limitations. It is a retrospective study of clinically collected data with risks of inaccurate or incomplete data. However, we ensured that the data were abstracted and audited using standard protocols to improve the reliability of the data. The data are from a hospital in southern California; thus, the results are not generalizable to other communities. Symptoms were self-reported and not specifically solicited thus increasing the possibility of underreporting. Also, the data analysis at the county level is skewed since approximately 92% of the subjects were from one county. We also did not report on contact tracing findings. Furthermore, there may be a selection bias since the majority of people tested were from San Bernardino County, thus the rates of COVID-19 infections reported for the other counties may not be truly reflective of the infection rates in those counties. However, we have included all the data to provide complete information about all patients tested at Arrowhead Regional Medical Center.

A strength of this study is that this is one of the few studies to focus only on risk factors for community spread which are important data required for public health planning to mitigate infection transmission. This study also studied the whole community as opposed to most previous studies that were either all entirely hospitalized patients or patients presenting to emergency departments or modeling of national or regional data. Additionally, we not only reviewed individual risk factors of the people tested but also the social and structural determinants of their respective cities of residence.

## Conclusions

Independent predictors of testing COVID positive in the Southern California community were Hispanics or primary Spanish speaking people, and those with diabetes or hypertension. Children 10 years or younger had the highest COVID-19 rate while males were more likely to test positive. COVID symptoms only occurred in approximately 33% with low sensitivity and specificity. Geographical variations of COVID-19 positive rates were accounted for by social and structural determinants of health. The data suggest that people who had a lack of resources, overcrowded housing, and deprived communities with higher rates of poverty and increased pollution levels were more likely to test COVID-19 positive. A greater understanding of the local factors in each community that contribute to the socio-economic variability in testing positive to COVID-19 will assist in the early identification of high-risk individuals and communities to enhance the precision of public health interventions.
